# Case series of perianal and pelvic MRI imaging findings in monkeypox

**DOI:** 10.1259/bjrcr.20220109

**Published:** 2022-09-26

**Authors:** Jason Gan, Janki Patel, Eleanor Ainsworth, Aatish Patel, Geraldine O'Hara, Ahmed Elowaidy

**Affiliations:** 1 Guys and St Thomas Hospital, London, United Kingdom

## Abstract

Monkeypox is a viral infection historically rarely seen in humans, but currently the focus of international attention due to a multi-country outbreak outside endemic countries of Central and West Africa, where cases are typically confined. Perianal pain and lesions have recently been recognised as a feature of monkeypox. We present a case series of the imaging findings of patients with monkeypox, including active proctitis, anal canal inflammation, and perianal inflammation. The aim is to increase awareness of perianal and rectal monkeypox MRI imaging features during this current outbreak.

## Introduction

Monkeypox is a viral infection historically rarely seen in humans, but currently the focus of international attention due to a multicountry outbreak outside endemic countries of Central and West Africa, where cases are typically confined.^1^ The World Health Organisation (WHO) has reported over 16,000 laboratory confirmed cases since this outbreak was recognised in May 2022, and case numbers are continuing to rise.^2^ At present for cases where sex is known 99% of cases are in males and within this group, 99% identify as gay, bisexual or males who have sex with males (GB MSM).^3,4^ Monkeypox is transmitted via close contact with skin lesions, body fluids, respiratory droplets, and contaminated materials such as bedding. Many patients report a mild course of illness with quick recovery, there are a subset of those who require hospitalisation. Symptoms associated with monkeypox include fever, headache, lymphadenopathy, fatigue, and a characteristic rash.^3^ Perianal pain, perianal lesions, and genital lesions have recently been recognised as a feature of monkeypox in this outbreak possibly related to close contact with lesions such as during sexual intimacy.^3^ Our centre has attended to a number of patients presenting with perianal symptoms, the severity of some has required admission and subsequent cross-sectional imaging to assess for complications. At the time of writing in July 2022, our centre has treated 113 confirmed cases of monkeypox, with 22 patients requiring admission.^3^

We present a case series of this subset of patients with monkeypox with their respective imaging findings with the aim of increasing awareness of perianal and rectal monkeypox MRI imaging features during this current multicountry outbreak.

## Case series

Written informed consent for the case to be published (incl. images and case history) was obtained from the patients for publication of this case report, including accompanying images.

All patients in this series are sexually active MSM in their 20–40 s. Any patient with concurrent inflammatory bowel disease (IBD) was excluded from this study due to overlapping imaging findings.

### Case 1

Patient presented with 1-week history of fever, sore throat, cough, and myalgia with severe perianal pain and bleeding per rectum. Examination revealed perianal vesicular rash and inflammation. Blood tests demonstrated normal white cell count and CRP of 47. No history of IBD or concurrent sexually transmitted infection.

#### Imaging (Figure 1)

MRI rectum study was performed, demonstrating mild active proctitis, mild inflammation of the lower perianal skin (corresponding to clinical perianal ulceration), and mildly enlarged mesorectal lymph nodes.

**Figure 1.**
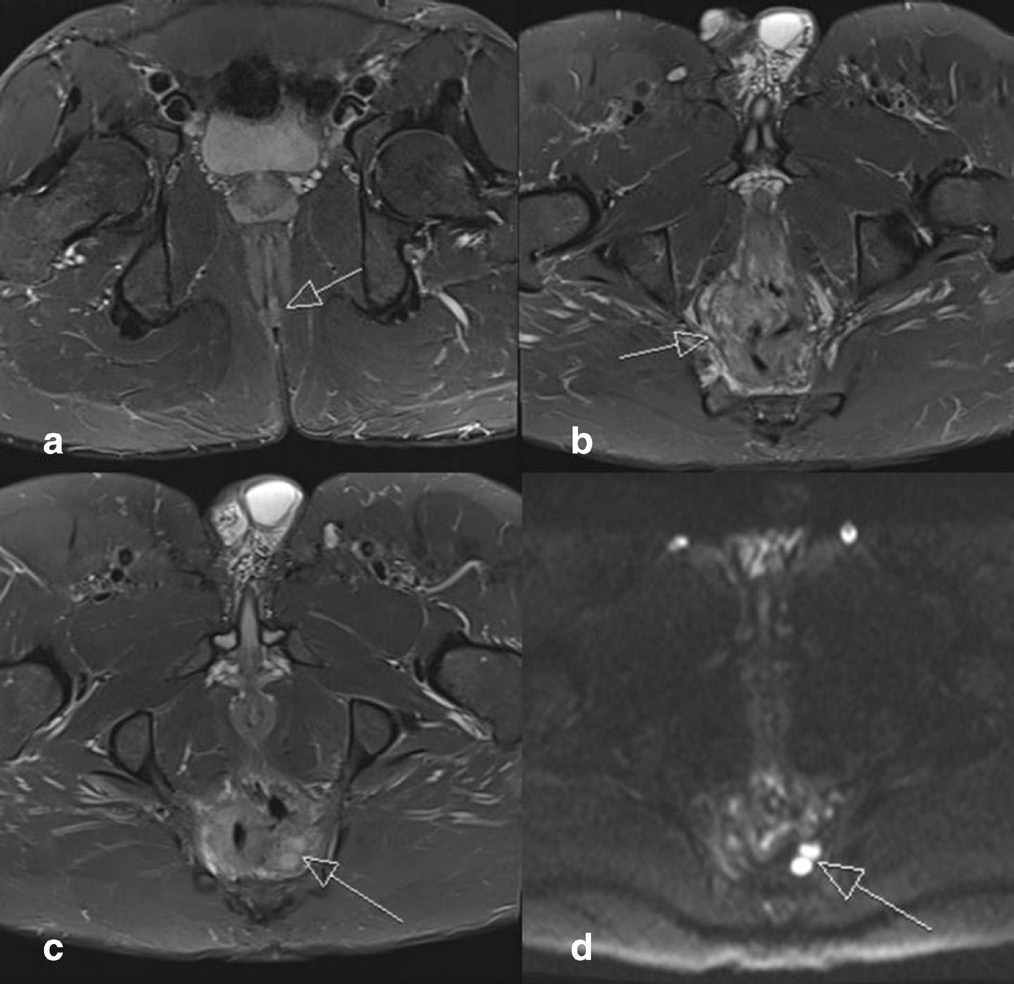
**A.** Axial STIR image demonstrates high signal around the anal verge at the perianal region in keeping with mild perianal inflammation (arrow). Figure 1B. Axial STIR image demonstrates high signal and thickening of the rectum in keeping with mild proctitis (arrow). Figure 1C. Axial STIR image demonstrates prominent mesorectal lymph nodes secondary to the adjacent proctitis. Figure 1D. Axial DWI image demonstrates corresponding restricted diffusion in the mesorectal lymph nodes.

**Figure 2.**
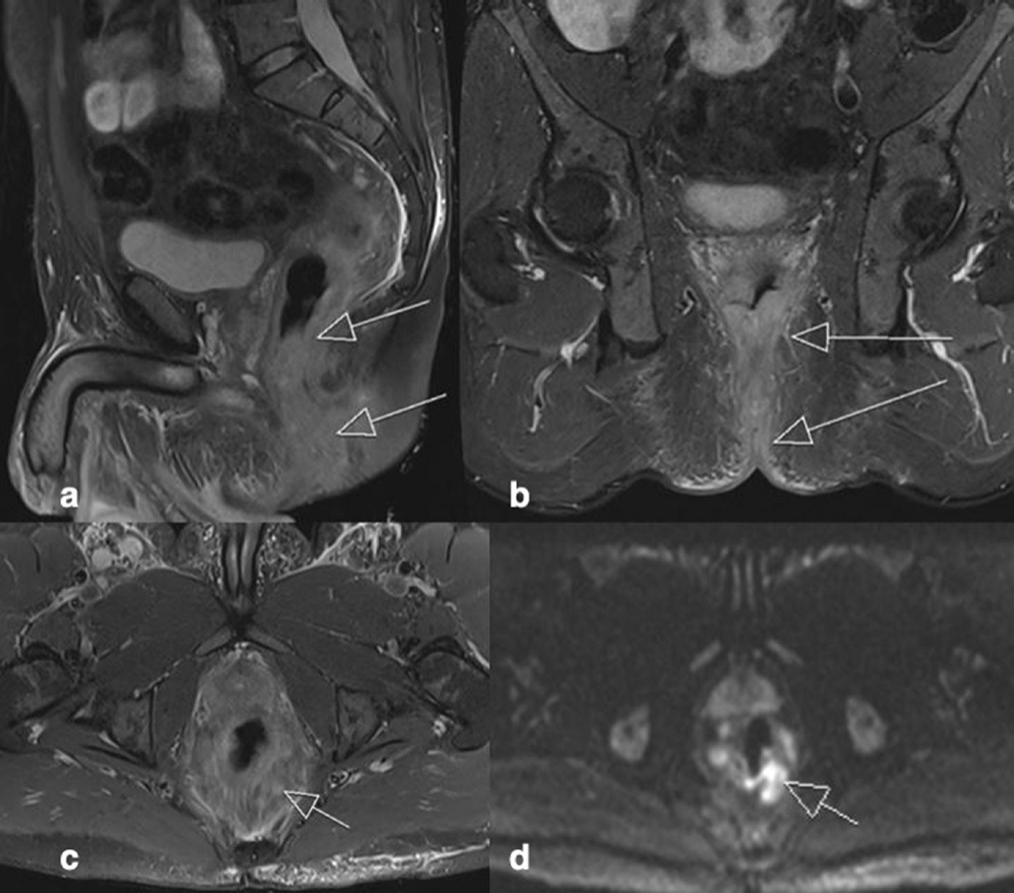
A. Sagittal STIR image demonstrates high signal of the rectum and anal canal in keeping with severe active proctitis and extensive inflammation of the anal canal (arrows). Figure 2B. Coronal STIR image demonstrates high signal extending from the anal canal to the perianal/perineal regions in keeping with extensive inflammation of perianal skin (arrows). Figure 2C. Axial STIR image demonstrates prominent mesorectal lymph nodes secondary to the adjacent proctitis. Figure 2D. Axial DWI image demonstrates corresponding restricted diffusion in the mesorectal lymph nodes.

### Case 2

Patient presented with fever, severe perianal pain, and inability to open his bowels. Examination revealed widespread erythematous rash with perianal oedema and induration and a deep perianal fissure at the 12 o’clock position. Blood tests demonstrated normal white cell count and CRP of 53. No history of IBD. The patient was HIV positive, on antiretroviral treatment with an undetectable viral load.

#### Imaging (Figure 2)

MRI rectum illustrated rectal and anal canal mural thickening with high T2/STIR signal and restricted diffusion in keeping with severe active proctitis and extensive active inflammation of the anal canal. The MR also showed extensive active inflammation of the perianal/perineal cutaneous and subcutaneous tissue. There was mesorectal inflammation and multistation lymphadenopathy.

### Case 3

Patient presented with fever, rash, and rectal pain resulting in inability to open bowels. On examination, cluster of white papules noted at the 3 o’clock position in the perianal region. Mildly raised CRP of 41 noted on blood tests, with normal white blood count. Previous medical history includes HIV on antiretroviral treatment with an undetectable viral load. No history of IBD.

#### Imaging (Figure 3)

A MRI rectum study performed during admission showed severe active proctitis, active inflammation of the upper anal canal mucosa, and left perineal/perianal cutaneous tissue with active inflammation. There was multistation lymphadenopathy.

**Figure 3.**
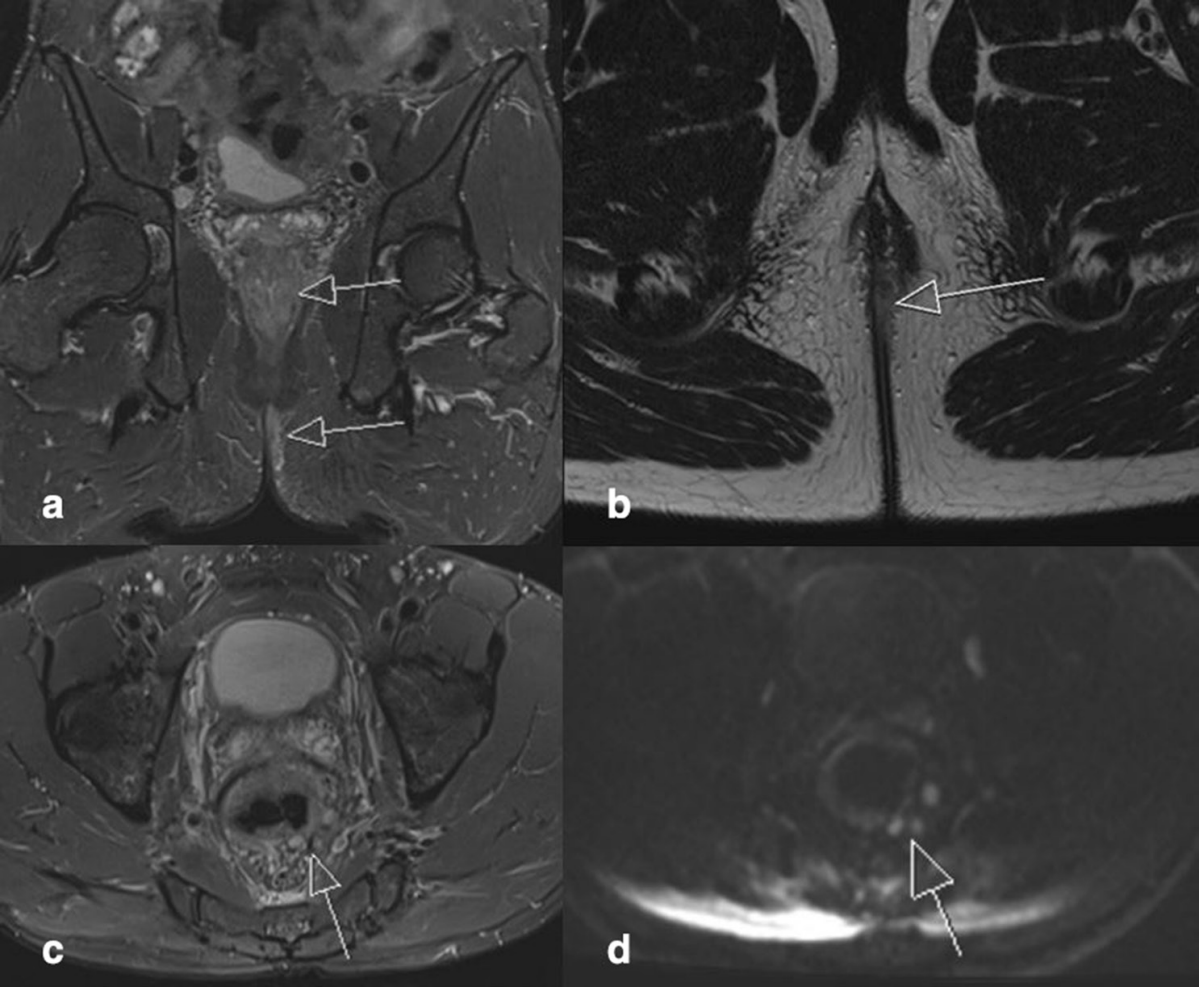
A. Coronal STIR image demonstrates high signal and mural thickening of the rectal wall with surrounding inflammation and mesorectal lymphadenopathy in keeping with severe active proctitis (superior arrow). There is also high signal in the perianal/perineal region in keeping with active inflammation of the left perianal region (inferior arrow). Figure 3B Axial T2 image demonstrates perineal/perianal active inflammation (arrow) Figure 3C. Axial STIR image demonstrates prominent mesorectal lymph nodes secondary to the adjacent proctitis. Figure 3D. Axial DWI image demonstrates corresponding restricted diffusion in the mesorectal lymph nodes.

All three cases demonstrated active proctitis, anal canal inflammation, and perianal inflammation, ranging from mild to severe (Table 1). Multistation lymphadenopathy was also seen in all cases. These radiological findings matched the clinical findings. These four features were also seen in another confirmed monkeypox MRI rectum; however, the case was excluded due to a history of IBD.

**Table 1.** Summary of MRI rectum findings.

	Proctitis	Anal Canal	Perianal	Other
**Case 1**	Mild	Mild inflammation of lower canal	Mild inflammation of perianal skin around the anal verge.	Mesorectal inflammation & mild local lymphadenopathy
**Case 2**	Severe	Extensive inflammation	Extensive inflammation of perianal skin on both sides	Mesorectal inflammation & extensive local lymphadenopathy
**Case 3**	Severe	Extensive inflammation	Mild inflammation of the perianal skin on the left side-only	Mesorectal inflammation & Extensive local lymphadenopathy

All patients were treated with antibiotics. Two patients in our case series were treated with Tecovirimat and analgesia. At the time of writing, all patients reported improvement of symptoms.

## Discussion

Recent publications and case studies have described the clinical manifestations of monkeypox, including genital^5,6^ and rectal^7^ involvement; however, there is limited information in the literature demonstrating the imaging findings in perianal and rectal monkeypox. To our knowledge, this is one of the very first case series addressing the imaging findings in pelvic and perianal monkeypox.

Proctitis and perianal inflammation have been described in association with sexually transmitted infections as far back as the 1980s^8^ with causative agents including *Neisseria gonorrhoeae*, *Chlamydia trachomatis*, herpes simplex virus, and *Treponema pallidum* (syphilis)^9,10^ or a combination of the above, as well as in isolated HIV infection.^11^ Human papillomavirus (HPV)-related perianal changes^12^ may mimic lesions secondary to monkeypox, and should be considered as a differential based on clinical assessment. There is notable overlap of some imaging findings in sexually transmitted infections with those of inflammatory BD^13^ and even rectal malignancy^14–18^ and this can lead to a delay in correct diagnosis and appropriate treatment. Moreover, some patients might present with more than one active STIs at the same time further complicating the imaging appearances.

Many sexually transmitted infections may present with non-specific active proctitis and anal canal inflammation, similarly to monkeypox. However, the pattern of the extensive inflammation of the perianal skin was a finding that was notable in our patients with monkeypox presented here compared to other STIs on imaging at our institution. This finding, of active perianal inflammation in the absence of perianal fistula or collection in the right clinical and epidemiological context, could be suggestive of, but not specific for monkeypox. Further case studies might be needed to assess this observation.

Furthermore, perianal fistula disease features have not been identified so far with monkeypox in our case series, as seen in IBD. There is, however, large overlap with monkeypox and other infectious/inflammatory causes of proctitis/perianal inflammation, highlighting the significance of clinical history, presentation, and investigations.

In the current climate, consideration of monkeypox in the differential diagnosis in male patients presenting with perianal and rectal pain, with imaging findings presented above would be prudent.

The small number of cases in our case series is a limitation of this study. Further case studies looking at the imaging findings of monkeypox are required to corroborate our findings.

## Learning points

- Monkeypox should be considered as a differential in cases of active proctitis, anal canal inflammation, and perianal inflammation, especially if the history is suggestive.
- Imaging features of monkeypox are non-specific, and there is overlap with some sexually transmitted infections, IBD, and rectal malignancy. The clinical history will be important in guiding the radiologist and clinician to the diagnosis.
- Perianal fistulous disease has not been documented as a feature of monkeypox, and if present, IBD should be considered as a differential.
